# Epidemiology of hepatitis D virus coinfection among HBsAg-positive individuals in Kermanshah, Western Iran

**DOI:** 10.3205/dgkh000648

**Published:** 2026-05-13

**Authors:** Niloufar Khodaei, Shahla Shahbazi, Mohsen Pirvesi, Mehdi Zobeiri, Babak Sayad, Nayebali Rezvani, Maria Shirvani

**Affiliations:** 1 Department of Microbiology, Faculty of Medicine, Iran University of Medical Sciences, Tehran, Iran; 2 Infectious Diseases Research Center, Health Policy and Promotion Institute, Kermanshah University of Medical Sciences, Kermanshah, Iran; 3 Clinical Research Development Center, Imam Reza Hospital, Kermanshah University of Medical Sciences, Kermanshah, Iran; 4 Department of Internal Medicine, School of Medicine, Kermanshah University of Medical Sciences, Kermanshah, Iran; 5 Reference of laboratory, Kermanshah University of Medical Sciences,Kermanshah, Iran

**Keywords:** Hepatitis B virus (HBV), Hepatitis D virus (HDV), coinfection, anti-HDV, ELISA, HBsAg-positive patients

## Abstract

**Background:**

Hepatitis delta virus (HDV) infection occurring in the presence of hepatitis B virus (HBV) is associated with severe liver disease, cirrhosis, and risk of liver cancer. Despite its clinical importance, data on the prevalence of HDV among HBV-infected individuals in western Iran, especially in Kermanshah, remain limited. This study aimed to determine the prevalence of HDV infection among HBsAg-positive patients using ELISA.

**Methods:**

This study included all HBsAg-positive patients referred to the Kermanshah Reference Laboratory during the first half of 2023. Demographic variables, liver enzymes, and HBV serological markers were collected. HDV-Ab testing was performed using commercial ELISA kits. Data were analyzed using SPSS 20, and statistical tests were applied to assess associations between HDV positivity and clinical or demographic variables.

**Results:**

A total of 284 HBsAg-positive patients were included. Mean ALT and AST levels were 27.2±41.8 U/L and 29.2±18 U/L, respectively. HBeAg and HBeAb were positive in 81% of patients. HDV-Ab positivity was observed in 12 individuals (4.2%). No significant associations were found between HDV infection and age, sex, ALT, AST, HBeAg status, or HBeAb status.

**Conclusion:**

The prevalence of HDV infection among HBsAg-positive patients was relatively low and showed no statistically significant association with demographic factors or routine laboratory markers.”We would also kindly request the same correction to be applied in the German abstract, replacing “keine Assoziationen” with “keine statistisch signifikanten Assoziationen” to accurately reflect the statistical analysis.

## Zusammenfassung

**Hintergrund:**

Eine Infektion mit dem Hepatitis-D-Virus (HDV), die in Anwesenheit des Hepatitis-B-Virus (HBV) auftritt, ist mit schweren Lebererkrankungen, Leberzirrhose und erhöhtem Risiko für Leberkrebs verbunden. Trotz der klinischen Bedeutung sind die Daten zur Prävalenz von HDV bei HBV-infizierten Personen im Westen Irans, insbesondere in Kermanshah, begrenzt. Ziel dieser Studie war es, die Prävalenz einer HDV-Infektion bei HBsAg-positiven Patienten mithilfe ELISA zu bestimmen.

**Methode:**

Es wurden alle HBsAg-positiven Patienten eingeschlossen, die im ersten Halbjahr 2023 an das Referenzlabor in Kermanshah überwiesen wurden. Es wurden demografische Variablen, Leberenzyme sowie serologische Marker einer HBV Infektion erhoben. Der Nachweis von Anti-HDV (HDV-Ab) erfolgte mit Hilfe kommerzieller ELISA-Kits. Die Daten wurden mit SPSS Version 20 analysiert, und statistische Tests angewendet, um Zusammenhänge zwischen HDV-Positivität und klinischen oder demografischen Variablen zu untersuchen.

**Ergebnisse:**

Es wurden 284 HBsAg-positive Patienten einbezogen. Die mittleren ALT- und AST-Werte betrugen 27,2±41,8 U/L bzw. 29,26±18 U/L. HBeAg und HBeAb waren bei 81% der Patienten positiv. Eine Anti-HDV-Positivität wurde bei 12 Personen (4,2%) festgestellt. Es zeigten sich keine signifikanten Zusammenhänge zwischen einer HDV-Infektion und Alter, Geschlecht, ALT, AST, HBeAg-Status oder HBeAb-Status.

**Schlussfolgerung:**

Die Prävalenz der HDV-Infektion bei HBsAg-positiven Patienten war relativ niedrig und zeigte keine Assoziationen mit demografischen Faktoren oder routinemäßigen Laborparametern. Angesichts der mit HDV assoziierten Lebererkrankungen und der Unfähigkeit biochemischer Marker, eine Koinfektion vorherzusagen, ist ein HDV-Screening bei HBV-infizierten Patienten essenziell. Das Verständnis der Faktoren, die die HBV-HDV-Koinfektion bestimmen, sowie regionaler Unterschiede in der HDV-Seropositivität kann zur Entwicklung wirksamer Strategien im Bereich der öffentlichen Gesundheit zur Prävention der Infektion beitragen.

## Introduction

Viral hepatitis is a global public health problem, affecting millions of people worldwide and leading to a wide array of clinical diseases due to chronic liver disease and acute liver failure [1], [2]. Among the main hepatotropic viruses numbered alphabetically (HAV, HBV, HCV, HDV, and HEV), the hepatitis B virus (HBV) is one of the most prevalent and clinically significant issues [3]. Unlike HAV and HEV, which are self-limited and transmitted by the fecal-oral route, HBV is transmitted through parenteral, perinatal, and sexual contact, leading to chronic disorder [4], [5]. 

Currently, HBV infection affects approximately 292 million people all around the world, causing around 887,000 deaths annually, mainly due to cirrhosis and hepatocellular carcinoma (HCC) [1], [6]. Although there are effective vaccines and treatment strategies, the drugs cannot eliminate the virus, and HBV infection remains a serious chronic liver problem, particularly in low- and middle-income countries [7]. Chronic HBV infection can result in cirrhosis, decompensated liver disease, and hepatocellular carcinoma. The course and outcomes are influenced by several factors, including age at infection, virus-host interactions, immune response, and co-infection with other viruses [8], [9].

hepatitis Delta virus (HDV) is a defective, minus single-stranded RNA of about 1,700 nucleotides which depends on HBV for replication and assembly [10], [11]. HDV is an obligate satellite virus requiring the HBsAg envelope proteins of HBV to form its viral particles [12]. More than 10 million individuals are struggling with HDV infection all around the world, and it is estimated that about 5% HBV-infected people are coinfected with HDV [13]. HDV infection occurs either as asymptomatic cases or acute liver failure and chronic hepatitis D (CHD), and it has been shown that cirrhosis, liver decompensation, and HCC progression are significantly faster among patients with chronic HDV infection compared with those infected with HBV alone [14], [15].

The prevalence of HDV infection varies widely across geographical regions and, in particular, is high in Africa, Latin America, Eastern Europe, the Middle East, and Central Asia [16], [17]. hepatitis D infection is reported in Iran, and prevalence rates differ between regions and populations [18]. Limited data are available on the frequency of HDV among HBV-infected individuals in western Iran, particularly in Kermanshah province. Therefore, this study aimed to investigate the prevalence of hepatitis D virus infection among HBsAg-positive patients in Kermanshah using the enzyme-linked immunosorbent assay (ELISA) method. 

## Materials and methods

### Study population and sampling

This descriptive cross-sectional study included all HBsAg-positive patients referred to the Kermanshah Reference Laboratory during the first half of 2023. To improve the study's accuracy, Census sampling was used, and all eligible patients presenting to the laboratory during the study period were enrolled. According to a study by Osiowy et al. [19] and the minimum sample size formula with a 95% confidence level, the required sample size was estimated at 262 participants. The study started after approval by the Ethics Committee and the Research Deputy of Kermanshah University of Medical Sciences (IR.KUMS.MED.REC.1402.340). Patient confidentiality was strictly maintained throughout the study. All collected data were anonymized before analysis to protect participants’ identities. Written informed consent was obtained from all patients prior to enrollment. The study was conducted in compliance with relevant ethical principles, and findings were reported in aggregated form, ensuring that no individual participant could be identified.

Access to patient laboratory records was granted, and a trained resident involved in the project extracted relevant demographic and clinical data.

### Serological testing (ELISA method)

Serum samples, obtained from 5 mL of venous blood routinely collected in EDTA-free tubes, were used for laboratory assessment. The variables evaluated in this study included demographic factors (age and sex) and laboratory parameters (ALT, AST, HBeAg, and HBeAb). Serum was separated and analyzed using commercial ELISA kits (RadimSpA, Italy) according to the manufacturer’s instructions. Briefly, serum samples were added to microplate wells that were pre-coated with specific antigens, incubated at room temperature, and then washed to remove any unbound material. An enzyme-linked secondary antibody (Radim S.p.A., Rome, Italy) was then applied, followed by the addition of TMB substrate (Radim S.p.A., Italy). After color development, the reaction was stopped, and absorbance was measured at 450 nm using an ELISA microplate reader (Stat Fax 4200, USA). Results were read based on the cut-off values provided by the kit manufacturers [20], [21].

### Statistical analysis

Data were collected, categorized, and analyzed using SPSS software version 20. Descriptive statistics, including mean, standard deviation, absolute frequencies, and relative frequencies, were used to summarize the findings.

## Results

### Demographic characteristics

A total of 284 HBsAg-positive patients who had been referred to the Kermanshah reference laboratory in the first half of 2023 were included. The mean age of the patients was 47.7±12.7 years, with most patients being in the age range of 30 to 60 years. 57% were male and 43% were female (Table 1 (Tab. 1)).

### Laboratory findings and viral markers

The mean ALT and AST levels were 27.4±19.8 U/L and 29.2±18U/L, respectively, with most patients showing values below 30 U/L. All patients were HBsAg positive. HBeAg and HBeAb positivity were both 81%, while HDV-Ab positivity was 4.2% (12 patients). HIV co-infection was observed in 3.2% (9 patients) (Table 2 (Tab. 2)).

### HDV prevalence according to study objectives

HDV prevalence was analyzed by age, sex, liver enzymes, and HBV serological markers. Kolmogorov-Smirnov tests confirmed that age, ALT, and AST did not follow a normal distribution (P<0.001). Statistical analyses showed that there were no significant differences between HDV-positive and HDV-negative patients in terms of age (P=0.624), sex (P=1.000), ALT (P=0.380), AST (P=0.152), HBeAg (P=0.410), or HBeAb (P=1.000) (Table 3 (Tab. 3)).

## Discussion

Among 284 HBsAg-positive patients the prevalence of HDV was 4.2% and the findings showed no significant difference between age, gender, and levels of liver enzymes ALT and AST. This may indicate that conventional clinical and biochemical indicators do not have sufficient ability to diagnose or predict HDV co-infection, and that specific screening remains necessary.

Comparison of the findings of this study with previous studies shows that the prevalence rate obtained in Kermanshah is lower than in some other Iranian provinces, such as Hamedan (17.3%) [22] and Khorasan Razavi (21.84%) [23], but it is similar to some global reports from countries, for instance, Canada (5%) [19]. Reports from African and Central Asian countries show higher prevalence (up to 15–20%) [17], [24], which is likely because of differences in behavioral patterns, epidemiological factors, type of sampling, and access to vaccination programs. The difference in HDV prevalence could be due to variations in high-risk groups, transmission modes, and differences in diagnostic tools used in the studies.

The lack of significant difference in mean ALT and AST between the two groups of infected and uninfected patients indicates that HDV infection is not always accompanied by a significant increase in liver enzymes, especially in the early stages or mild forms of the disease. Therefore, relying solely on biochemical tests to identify HDV cases is insufficient; specific serological or molecular tests are essential for early diagnosis. This finding highlights the importance of screening of HBV-positive patients, especially those with high-risk behaviors, dialysis patients, or those with unknown liver problems.

The strengths of the present study include the use of real-world data from a reference center and a standard diagnostic method, which increases the validity of the results. 

## Limitations

The study had some limitations. This study is a cross-sectional one, which causes a limitation in the evaluation of cause-and-effect relationships. Moreover, there is a lack of viral RNA testing to confirm active infection. The relatively small number of HDV-positive patients is another problem in the current study, which limited comparative analyses. Therefore, larger studies with a greater design and the use of molecular methods could provide a more accurate understanding of the true burden of the disease and its long-term consequences. 

## Conclusion

Although the prevalence of HDV infection in our cohort of HBsAg-positive patients was low and showed no significant association with demographic or laboratory variables, recognition of the factors contributing to HBV and HDV coinfection and understanding the causes of variations in positive anti-HDV among HBV patients is essential for healthcare managers and policymakers to manage and limit this public health concern effectively.

## Notes

### Authors’ ORCIDs

- Khodaei N: https://orcid.org/0009-0002-6303-4446
- Shahbazi S: https://orcid.org/0000-0001-6325-1075
- Pirvesi M: https://orcid.org/0009-0009-1440-0236
- Zobeiri M: https://orcid.org/0000-0002-9394-8399
- Sayad B: https://orcid.org/0000-0001-8686-9986
- Rezvani N: https://orcid.org/0000-0001-6153-0345
- Shirvani M: https://orcid.org/0000-0003-3690-2594

### Ethical approval 

The study was approved by Kermanshah University of Medical Sciences (approval code: IR.KUMS.MED.REC.1402.340). 

### Funding

None. 

### Acknowledgments

The study was supported by Kermanshah University of Medical Sciences.

### Competing interests

The authors declare that they have no competing interests.

## Figures and Tables

**Table 1:**
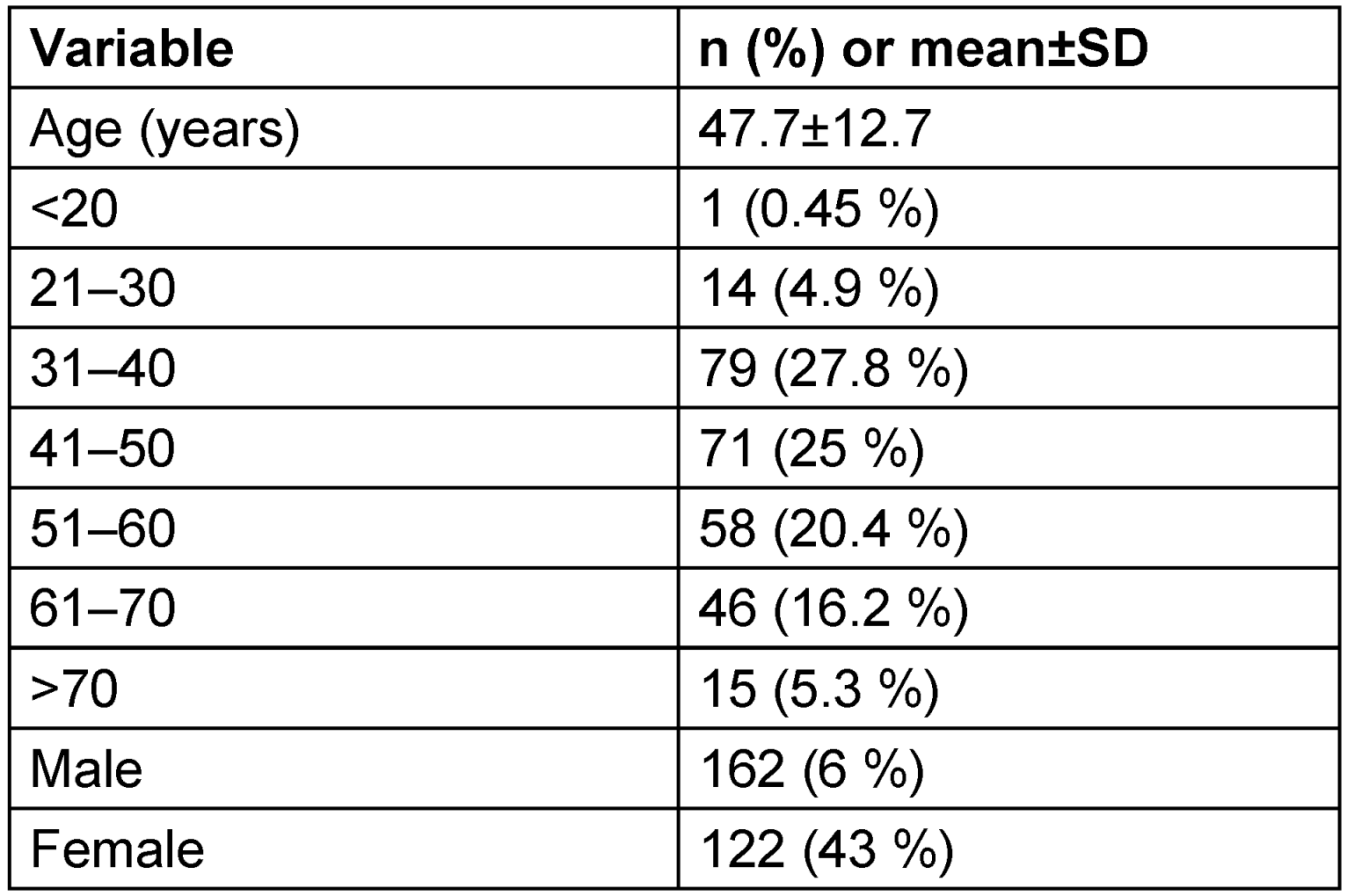
Demographic characteristics of patients

**Table 2:**
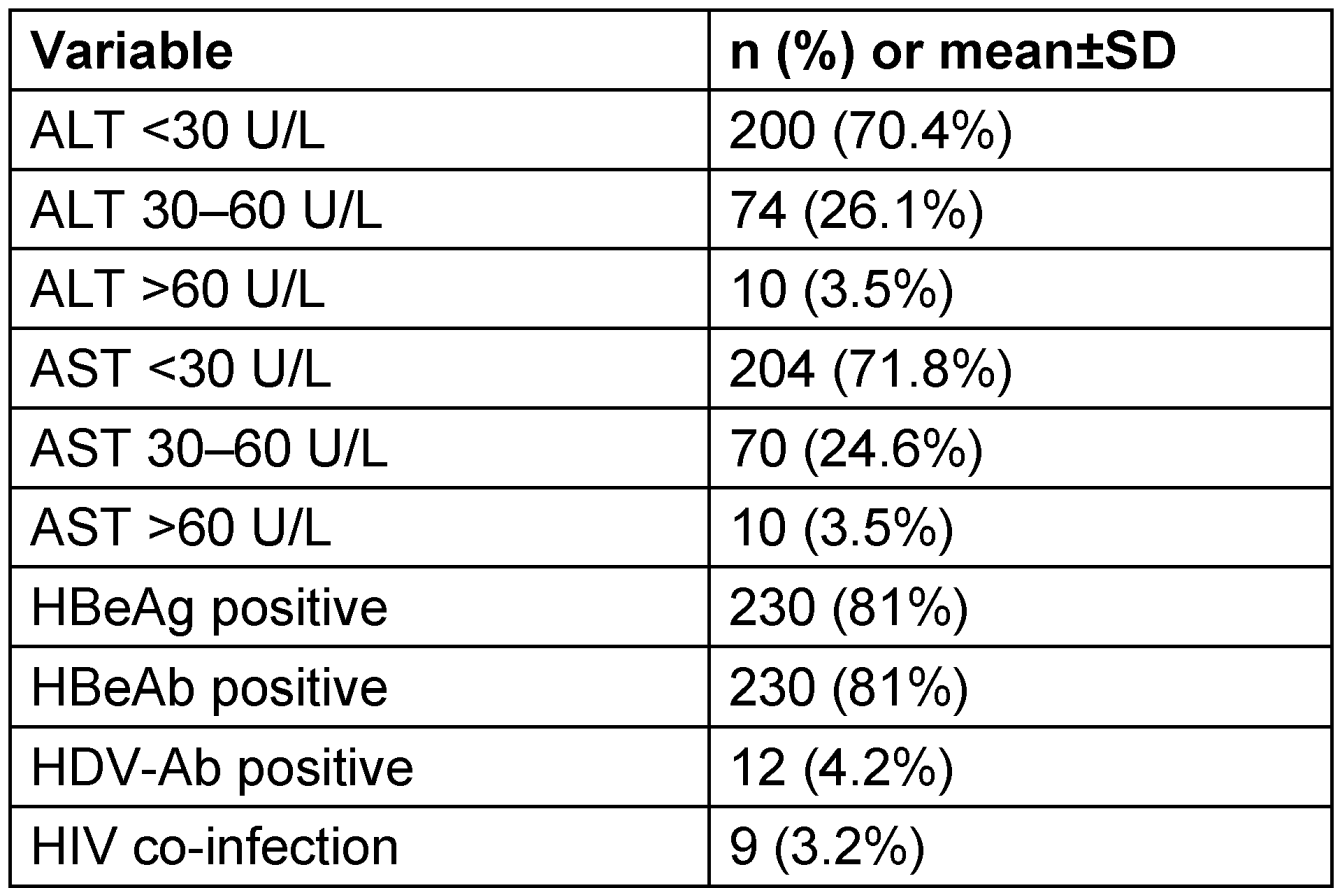
Laboratory findings and viral markers

**Table 3:**
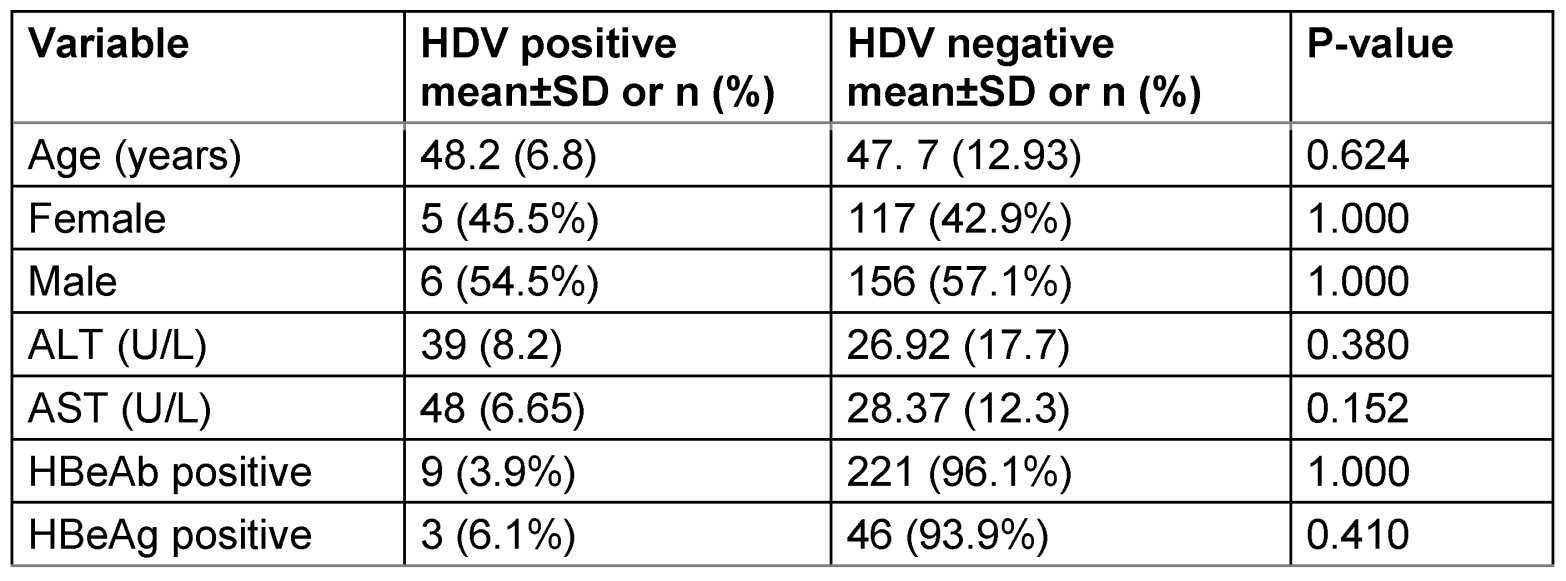
HDV prevalence according to age, sex, liver enzymes, and HBV markers
